# Metabolic Pattern of the Acute Phase of Subarachnoid Hemorrhage in a Novel Porcine Model: Studies with Cerebral Microdialysis with High Temporal Resolution

**DOI:** 10.1371/journal.pone.0099904

**Published:** 2014-06-18

**Authors:** Christoffer Nyberg, Torbjörn Karlsson, Lars Hillered, Elisabeth Ronne Engström

**Affiliations:** 1 Department of Neuroscience, section of Neurosurgery, Uppsala University, Uppsala, Sweden; 2 Department of Surgical Sciences, section of Anesthesiology and Intensive care, Uppsala University, Uppsala, Sweden; Glasgow University, United Kingdom

## Abstract

**Background:**

Aneurysmal subarachnoid hemorrhage (SAH) may produce cerebral ischemia and systemic responses including stress. To study immediate cerebral and systemic changes in response to aneurysm rupture, animal models are needed.

**Objective:**

To study early cerebral energy changes in an animal model.

**Methods:**

Experimental SAH was induced in 11 pigs by autologous blood injection to the anterior skull base, with simultaneous control of intracranial and cerebral perfusion pressures. Intracerebral microdialysis was used to monitor concentrations of glucose, pyruvate and lactate.

**Results:**

In nine of the pigs, a pattern of transient ischemia was produced, with a dramatic reduction of cerebral perfusion pressure soon after blood injection, associated with a quick glucose and pyruvate decrease. This was followed by a lactate increase and a delayed pyruvate increase, producing a marked but short elevation of the lactate/pyruvate ratio. Glucose, pyruvate, lactate and lactate/pyruvate ratio thereafter returned toward baseline. The two remaining pigs had a more severe metabolic reaction with glucose and pyruvate rapidly decreasing to undetectable levels while lactate increased and remained elevated, suggesting persisting ischemia.

**Conclusion:**

The animal model simulates the conditions of SAH not only by deposition of blood in the basal cisterns, but also creating the transient global ischemic impact of aneurysmal SAH. The metabolic cerebral changes suggest immediate transient substrate failure followed by hypermetabolism of glucose upon reperfusion. The model has features that resemble spontaneous bleeding, and is suitable for future research of the early cerebral and systemic responses to SAH that are difficult to study in humans.

## Introduction

The rupture of an intracranial aneurysm leads to transmission of blood with arterial pressure into the subarachnoid space, creating subarachnoid hemorrhage (SAH). This results in elevation of intracranial pressure (ICP), decrease of cerebral perfusion pressure (CPP) and transient global brain ischemia that in turn generates a cascade of secondary events. One consequence of aneurysmal SAH that has been reported is a stress response, including the release of ACTH, cortisol and catecholamines. [1], [2] The benefit of this hormonal response may be increased systemic blood pressure and a restored cerebral blood flow. On the other hand, systemic stress load is potentially harmful. Myocardial infarction [3] and acute myocardial failure are frequent complications of SAH, and may in turn cause neurogenic pulmonary edema as well as release of natriuretic peptides from the cardiac wall. [4], [5] These peptides increase sodium and water losses, rendering the patients hypovolemic. There is an increasing interest of better understanding the immediate consequences of the bleeding and the early brain injury, but in general, patients are transferred to a neurosurgical unit in a secondary hospital and there is a considerable delay before neuro-ICU care is provided with adequate monitoring.

Therefore, these very early events have to be studied in animal models of acute spontaneous SAH. The animal models described in the literature [6]–[11], were usually designed for research questions targeting later occurring intracranial events. Rats have been used frequently, but it can be questioned how well the rat physiology applies to human conditions. It has been shown that rats are resistant to glucose depletion, even during a 50 minute period of brain hypoxia [12], suggesting that the cerebral energy metabolism of a rat is different from the human metabolism, and possibly more efficient. Rabbits have also been used in SAH models [8], [9]. However, since both the rat and the rabbit are lissencephalic animals, they have smaller relative volume of the cortex compared to humans. We wanted to explore a model in which we could perform studies of both intracranial and systemic changes in the very acute stage of SAH. This required a robust animal with a physiology resembling human conditions. The larger relative neuronal volume of a gyrencephalic brain like that of a pig may render it more sensitive to ischemia and generally closer resembling a human brain in terms of energy metabolism. Also, we have previous good experiences from using the pig as an animal model for studies of systemic changes in other conditions. [13] The pig is an experimental animal that offers several advantages compared to smaller animals. Since the pig is a relatively large animal, it is easy to study and monitor vital parameters. The larger size also makes collection of samples of blood, urine and other body fluids with lower risk of hypovolemia and anemia as a consequence.

The aim of the present study was to develop a pig model of SAH with the focus on the effects of the initial impact on cerebral hemodynamics and brain energy metabolism. Specific for our model is that we tried to mimic the conditions at an aneurysm rupture by injecting blood in the basal cisterns until the cererbal perfusion pressure was zero, creating a transient global brain ischemia. The impact on ICP, CPP and brain energy metabolism measured with intracerebral microdialysis is described, as well as findings on computed tomography (CT) and post-mortem brain tissue examination.

## Material and Methods

### Ethics

The study protocol was approved by the Uppsala Institutional Review Board for Animal Experimentation (Permit number C187/11). The pigs were handled according to the guidelines of the Swedish National Board for Laboratory Animals and the European Convention of Animal Care.

### Animals and Anesthesia

The study was performed on a series of 11 pigs. The animals were of mixed breed and of both sexes. The age of the pigs was 8–10 weeks with a mean weight of 24.9 (23.1–28.8) kg. At induction of anesthesia, the animals received a bolus fluid infusion with 30 mL/kg of Ringer-acetat (Fresenius Kabi, Uppsala, Sweden). Thereafter, fluid infusion was administered with Ringer-acetat at 8 mL/kg/h and Rehydrex 25 mg/mL (Fresenius Kabi) at 10 mL/kg/h. Before transportation to the laboratory, the animals were given intramuscular injections of 50 mg xylazine (Rompun vet, Bayer Health Care, Leverkusen, Germany).

Induction of anesthesia was performed with intramuscular injections of tiletamine 3 mg/kg and zolazepam 3 mg/kg (Zoletil 100, Virbac, Carros, France), xylazine 2.2 mg/kg (Rompun vet, Bayer Health Care) and atropine 0.04 mg/kg (Atropin Mylan, Mylan, Stockholm, Sweden) in combination with intravenous injections of ketamine 100 mg (Ketaminol vet, Intervet International, Boxmeer, Netherlands) and morphine 1 mg/kg (Morfin Meda, Meda, Solna, Sweden). Maintenance of anesthesia was achieved by continuous intravenous infusion of ketamine 20 mg/kg/h, morphine 0.5 mg/kg/h and rocuronium bromide 2 mg/kg/h (Esmeron, NV Organon, Oss, Netherlands).

Immediately after the experiment was finished (140 minutes after induction of SAH), the animals were euthanized with potassium chloride while still under anesthesia. Thereafter either a computed tomography scan or morphological examination of the brain was performed.

### Preparation

The animals were tracheotomized and mechanically ventilated. The arterial partial pressure of carbon dioxide was kept within 5.0–6.0 kPa. The inspired gas was set to contain 30% oxygen mixed with air. A central venous catheter and a pulmonary artery catheter were inserted. A cervical branch from the subclavian artery was used to introduce an arterial catheter. A suprapubic catheter was placed directly in the bladder to deviate urine. The body temperature was monitored and kept within normal limits (37–39°C) throughout the experiment.

A midline incision was made on the skull; the scalp tissue and the periostium were removed from the bone to visualize the coronal suture. One burr hole was made 1 cm left of the midline and 1 cm anterior to the coronal suture. A corresponding burr hole was made on the right side. In the left burr hole, an external ventricular drain (EVD) stylet was inserted and placed on the anterior skull base aiming to get the tip in the midline. A catheter without side holes was inserted over the stylet to the skull base. The stylet was removed and the catheter was connected to a stopcock. The right burr hole was used to insert an intra-parenchymatous ICP monitor (Integra Camino, Integra Neurosciences, Plainsboro, NJ) that was secured with a screw-bolt into the skull bone.

About 2 cm anterior to the right burr hole, a third hole was made in which a microdialysis catheter (70 Brain Microdialysis Catheter, membrane length 10 mm, M Dialysis AB, Solna, Sweden) was inserted into the brain parenchyma. The catheter was connected to a 107 Microdialysis pump (M Dialysis AB) with a flow rate set at 1 µL/min with Perfusion fluid CNS (M Dialysis AB). The burr holes were sealed with bone wax to prevent leakage of cerebrospinal fluid. Microdialysate was analyzed for glucose, lactate and pyruvate using a CMA 600 bedside analyzer or ISCUSflex Microdialysis Analyzer (M Dialysis AB). The correlation between CMA600 and ISCUSflex data from separate runs with the same control sample was found to be excellent (for all analytes used here the *r* was >0.987) allowing for direct comparison of data from both instruments. Both analyzers were automatically calibrated when started as well as every sixth hour using standard calibration solutions from the manufacturer (M Dialysis AB). Imprecision values for between assay coefficient of variation was <10% for all analytes.

The position of the catheter on the skull base was verified either with fluoroscopy, CT after the experiment or post-mortem brain tissue examination.

### SAH Induction

After preparation and starting of microdialysis collection, at least 15 minutes of baseline data was collected before induction of SAH.

Autologous blood was injected through the catheter placed on the anterior skull base. The blood was aspirated from a central venous catheter or from an arterial catheter immediately before induction of SAH. The blood was injected slowly during 1–2 minutes with a 20 mL syringe, while monitoring ICP closely. The injection continued until CPP was kept around 0 for at least one minute. ICP and CPP was then allowed to recover spontaneously.

### Data Collection and Statistics

The first 30 minutes after microdialysis catheter insertion, the collected dialysate was discarded, and thereafter microdialysate was collected in fractions of 5 minutes. The collected dialysate was analyzed for concentrations of glucose, pyruvate, lactate and urea; the lactate-pyruvate ratio was calculated. Urea was monitored to control probe performance. [14] Dead space in the microdialysis collection system and in vivo extraction efficiency (relative recovery) were not compensated for. Relative recovery *in vivo* for glucose, pyruvate and lactate with the microdialysis catheter and perfusion rate used here is typically 20–30% in human brain [15]. The corresponding normal values in human brain (mean±SD) are for glucose, 1.2±0.6 mmol/L; pyruvate, 70±24 µmol/L; and lactate, 1.2±0.6 mmol/L [16].

Monitoring data containing arterial pressure (AP) and intracranial pressure (ICP) was collected and recorded with BIOPAC AcqKnowledge 3.9.1.6 (BIOPAC Systems, Goleta, CA). Cerebral perfusion pressure (CPP) was calculated using the formula CPP = AP-ICP.

Data and microdialysate were collected for 135 minutes after SAH induction.

Statistical analyses were performed with the software STATISTICA 10 (StatSoft, Inc. Tulsa, OK). The change of AP, ICP and microdialysis concentrations were evaluated with the Wilcoxon matched pairs test. Values of p<0.05 were considered to be significant.

## Results

### Arterial and Intracranial Pressures

**Figure 1:**
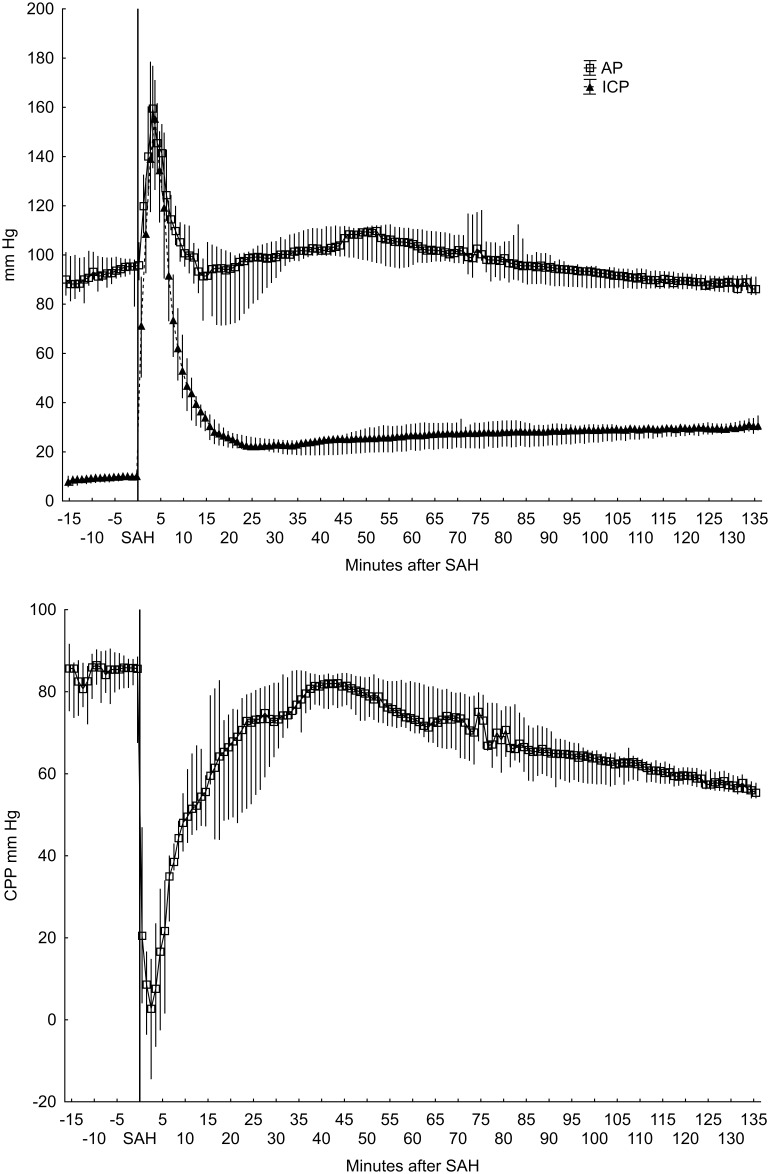
Arterial, intracranial and cerebral perfusion pressures in animals without persisting ICP elevation (n = 9). Median values with 25th–75th percentiles. AP and ICP (upper), CPP (lower).

The amount of blood injected varied from 13–25 ml. ICP typically increased to around 200 mm Hg (Figure 1). The AP increased shortly after the elevation of ICP. The rise of AP and drop in CPP was very rapid, peaking at the time when the injection of blood stopped. In some of the animals, the elevation of ICP was accompanied by extension posturing as well as cardiac arrhythmia or agonal breathing pattern.

After the peak in ICP at time of SAH induction, ICP levels slowly decreased to close to normal levels, but were still elevated compared to baseline values. In nine animals, ICP levels thereafter slowly increased during the remainder of the study time. In these animals, AP before induction of SAH had a mean value of 91 (70–106) mm Hg, compared to the peak value during SAH induction of 176 (140–210) mm Hg (p = 0.0077), and the AP at the termination of the experiment of 87 (74–92) mm Hg (p = 0.074). ICP before SAH had a mean value of 9 (2–13) mm Hg, a peak value of 202 (122–267) mm Hg (p = 0.0077), and the end value was 30 (20–34) mm Hg (p = 0.012).

In the remaining two animals, ICP remained elevated throughout the study time. CPP also remained negative after SAH induction. In these animals, microdialysis data also indicated severe persisting brain ischemia after SAH induction, as described below.

### Microdialysis

**Figure 2:**
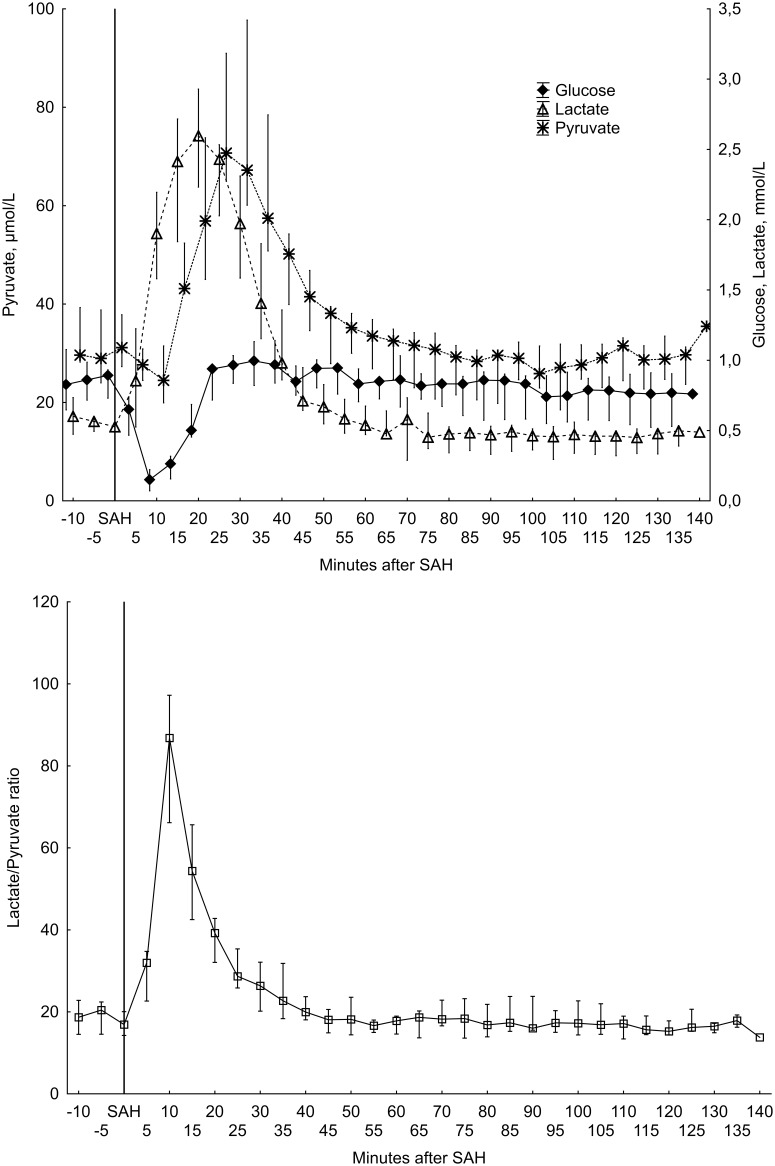
Microdialysis results from the animals without persisting ICP elevation (n = 9). Median values with 25th–75th percentiles. Energy related metabolites (upper) and Lactate/Pyruvate ratio (lower).

The analysis of the collected microdialysate showed two different patterns. Nine animals had a similar pattern in which glucose concentrations in the cerebral parenchyma dropped quickly after SAH induction (Figure 2). Glucose before SAH had a mean concentrations of 0.84 (0.51–1.13) mmol/L and decreased to 0.17 (0.02–0.45) mmol/L (p  = 0.012). Pyruvate levels initially also decreased, but then rapidly increased; mean pyruvate concentration before SAH was 32.9 (22.2–50.9) µmol/L and peaked at 25 minutes at 72.7 (37.5–105.8) µmol/L (p = 0.0077). Lactate concentrations increased rapidly after onset of SAH from 0.59 (0.51–1.13) to 2.6 (1.1–4.2) mmol/L (p = 0.0077) at 20 minutes. Consequently, the lactate-pyruvate ratio peaked in microdialysate collected 5–10 minutes after induction of SAH; initial lactate –pyruvate ratio had a mean value of 17.5 (13.3-23-5) and peaked at 89.9 (46.6–168.2) at 10 minutes (p = 0.0077). After these initials peaks, all analyzed metabolites slowly returned to concentrations similar to those collected from baseline data. The dead space in the microdialysis collection system is 5.1 µL, meaning that the microdialysate results reflects conditions 5.1 minutes earlier than the time of collection since the flow rate is 1 µL/min.

**Figure 3:**
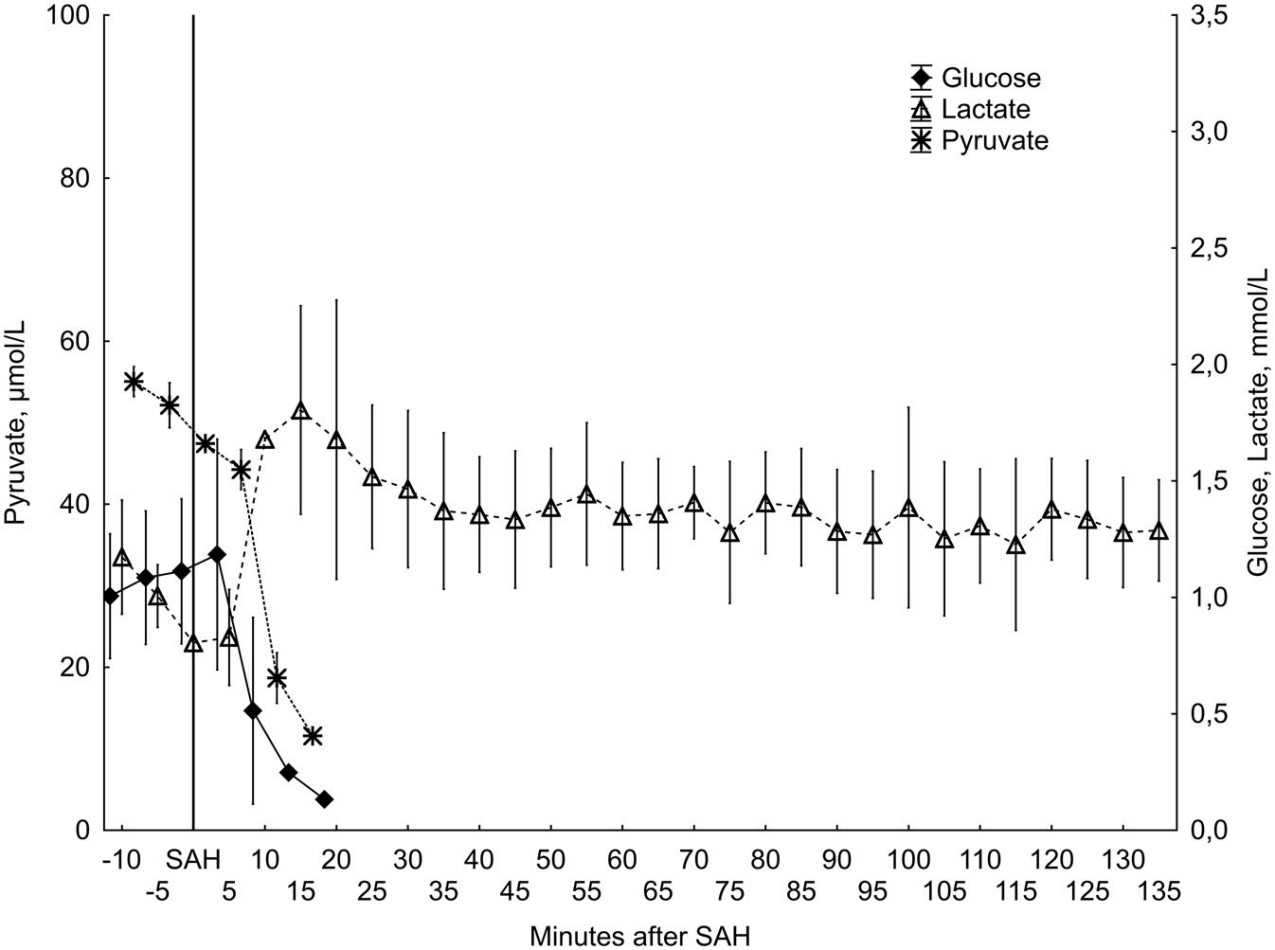
Microdialysis results from animals with persisting ICP elevation (n = 2). Mean values with minimum and maximum values.

In the two animals described above with persistent elevation of ICP, a typical microdialysis pattern of severe ischemia was seen. [17], [18] In these cases, glucose and pyruvate dropped quickly to undetectable levels and remained at those levels throughout the study time. At the same time, lactate concentrations increased and remained elevated (Figure 3).

### CT and Post-mortem Morphological Examination

**Figure 4:**
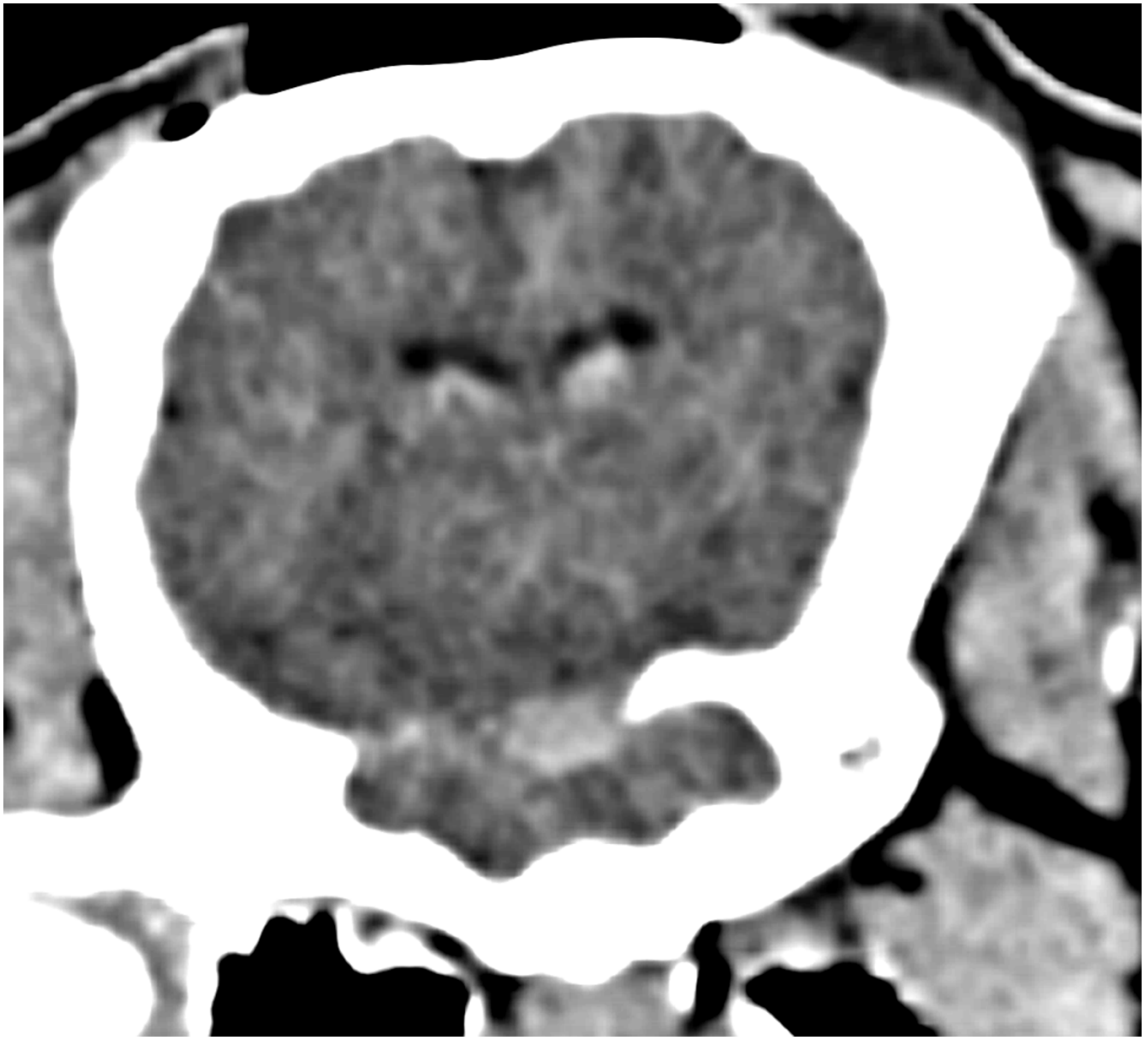
CT image after experiment verifying blood distribution in the basal cisterns. Blood is also visible in the lateral ventricles. Image width 7

The post-mortem examinations and the CT scans showed blood distribution in the basal cisterns in all animals. An example of the typical CT image is shown in Figure 4. In four animals, small amounts of coagulated blood were also found in the lateral ventricles. In one animal, there was a larger intra-parenchymatous hematoma. This animal was also one of the two animals with persisting high ICP and ischemic pattern in microdialysis.

## Discussion

Studies of acute metabolic, hormonal and hemodynamic changes after SAH require an animal model. There are multiple existing SAH models in different species and with different advantages. However, most models are focused on delayed vasospasm and events later in the course of SAH. We decided to develop an animal model in the pig with focus on the very early events after SAH. We consider the pig to be easy to monitor and study using the same equipment as in a clinical situation.

The pathophysiology of a SAH is complicated and can be viewed from different perspectives. The most important issue is probably the creation of the transient global ischemia. In order to do this in a reliable way, ICP and CPP need to be monitored. This has been described for animal models using rabbits [8] or rats. [7], [19] One advantage with the model in this study was that the severity of the SAH could be controlled during induction since ICP, CPP and AP were monitored in real-time. We verified the ictal global ischemia by recording a transient negative CPP. Since we targeted ICP and CPP, different amounts of blood were injected. This probably correlates to individual variations in the volume of the intracranial extracerebral space. In our protocol we decided to keep CPP zero for approximately one minute. We based this on our own experience from patients. It is common that patients are admitted in a fairly good clinical condition after a brief period of unconsciousness, presumably caused by transient brain ischemia at the aneurysm rupture. This is a challenging patient group since the clinical course can be quite unpredictable with some patients deteriorating while others have a good outcome. When the blood injection in our model stopped, ICP and CPP gradually returned to pre-injury levels spontaneously. However, during the measurements following SAH, ICP slowly increased. This elevation of ICP may be interpreted as the development of hydrocephalus due to the SAH, as frequently seen in patients.

The ischemia was further evaluated by intracerebral microdialysis (MD) for energy related biomarkers. The finding in our model was an immediate transient ischemic MD pattern with low glucose, high lactate and slightly decreased pyruvate, causing a markedly elevated lactate/pyruvate ratio. This energy metabolic biomarker pattern of cerebral ischemia has been validated in a number of studies, using e.g. PET. [17], [18] Interestingly, this short-lasting ischemia was followed by a period of elevated lactate and pyruvate and peaking at 15–25 minutes (20–30 minutes without compensation for dead space) after SAH in conjunction with normalized glucose. The peaks in lactate and pyruvate were not simultaneous, pyruvate peaking 5 minutes after lactate. This pattern may reflect a post-ischemic hyperemia allowing glucose and oxygen return with an ensuing hyperglycolytic state to produce energy for restoring ionic perturbation following the ischemic event. From about 45 minutes after SAH and throughout the remaining monitoring time, all analyzed MD energy-related biomarkers were normalized.

Previous studies have demonstrated alterations in the cerebral energy metabolism after SAH [19]–[21]. However, in these studies, since MD collection was performed at intervals of 15–30 minutes, the delay in the pyruvate peak compared to lactate was not detected. MD data with high temporal resolution at the time of bleeding has to our knowledge not been published before. A delayed pattern of hyperglycolysis has been observed in SAH patients and associated with favorable outcome, although those measurements were not done in the ultra-early phase. [22], [23] One problem with microdialysis is that it provides a focal measurement. However, in the present model we assumed that the change in CPP following the intervention produced global energy metabolic perturbation, presumably making our microdialysis data representative.

Another important matter in order to simulate the patient situation is the distribution of the blood. In the few published studies on SAH models in pigs, [6], [24]–[27] blood was injected in the cisterna magna. However, prechiasmatic blood location, as in the present model, may be important, especially when studying systemic effects, due to its proximity to the hypothalamus/pituitary systems. Prechiasmatic blood deposit has only been used in a small number of rat studies. [20], [27], [28] Injection of blood through a catheter placed on the frontal skull base has not been described before. The blood distribution in the animals resembles the distribution found clinically in patients and described in the Fisher scale [29], with blood in the basal cisterns, and sometimes in the ventricles or occasionally in the parenchyma.

Finally, blood degradation products are also important for the pathophysiology of SAH, and are involved in the development of vasospasm, inflammatory processes and metabolic changes [30]. However, these effects come after the time window we studied.

Clinically several of the animals displayed extension posturing, agonal breathing pattern or cardiac arrhythmias, thus resembling the situation of brain stem ischemia in patients. The fact that two out of eleven animals did not recover from the impact after SAH also reflects the situation in patients, although in an experimental study, this fact may not be beneficial. These facts combined with the findings of cerebral microdialysis and ICP/CPP demonstrate that the pig model described in this study is a good SAH model for acute studies of early events.

### Limitations of the Study

A confounding factor in this model was that all animals were mechanically ventilated and received anesthetic agents. This probably reduced the risk of acute death or prolonged ischemia at the time of SAH induction and provided more favorable conditions in the model compared to the situation in humans at the time of aneurysm rupture. We decided to use ketamine for maintenance of anesthesia to avoid the significant reduction of cerebral metabolism often associated with anesthetics.

The disadvantages of using the pig in an animal model rather than rodents include higher costs for the animals and more resources for conducting the experiment, thus limiting the number of animals used. However, we believe that the pig’s similarities to humans may make it superior in some aspects in studies of SAH.

## Conclusions

A novel pig model has been developed for studies of the acute stage of SAH. This study demonstrates that the model is representing features of aneurysmal bleeding seen in patients, with respect to ICP/CPP, energy metabolic changes, radiology and clinical expression. Since the pig is a larger animal than used in most SAH models, allowing for more extensive monitoring and sample collection, it seems well suited for future studies of early cerebral and systemic changes following SAH. The microdialysis pattern of energy metabolites with high temporal resolution at the ultra-early phase after SAH indicates that these events are not yet fully understood and should be further studied.
